# *QuickStats*: Age-Adjusted Death Rates* Attributable to Alcohol-Induced Causes,^^†^^ by Race/Ethnicity — United States, 1999–2015

**DOI:** 10.15585/mmwr.mm6618a12

**Published:** 2017-05-12

**Figure:**
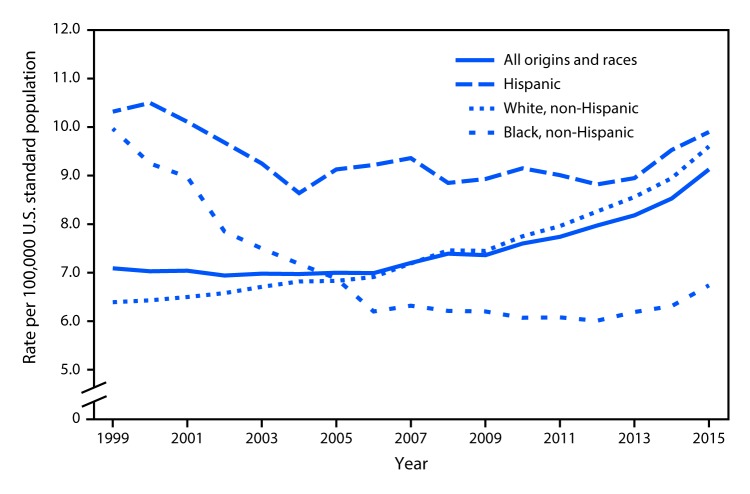
In 2015, mortality from alcohol-induced causes reached the highest rate during 1999–2015 of 9.1 deaths per 100,000 U.S. standard population. Alcohol-induced death rates for the Hispanic population remained the highest (9.9 per 100,000 U.S. standard population), followed by the non-Hispanic white population (9.6). For the non-Hispanic black population, the alcohol-induced death rate decreased 33% from 1999 to 2015, while the rate increased by 50% during the same period for the non-Hispanic white population. Overall, from 1999 to 2015, mortality from alcohol-induced causes increased 28% (7.1 to 9.1).

**Source:** National Vital Statistics System. Mortality public use data files, 1999–2015. https://www.cdc.gov/nchs/data_access/vitalstatsonline.htm.

